# Adverse drug reaction (ADR) monitoring of antihypertensive drugs in a tertiary care setting: A prospective observational study

**DOI:** 10.6026/973206300214133

**Published:** 2025-11-15

**Authors:** Mehre Darakhshan Mehdi, Md. Imran Mehdi, Mehre Afshan Mehdi, Abdullah Md. Umar, Zohra Jabeen

**Affiliations:** 1 Department of Pharmacology, ESIC Medical College and Hospital, Bihta, Patna, Bihar, India; 2 Department of Anaesthesia, Saroja Sitaram Sadar Hospital, Sheohar, Bihar, India; 3 Department of Obstetrics and Gynaecology, Nalanda Medical College and Hospital, Patna, Bihar, India; 4 Department of Surgery, Patna Medical College and Hospital, Patna, Bihar, India; 5 Department of Obstetrics and Gynaecology, Saroja Sitaram Sadar Hospital, Sheohar, Bihar, India

**Keywords:** Adverse drug reaction, pharmacovigilance, antihypertensive agents, hypertension, drug safety, naranjo algorithm

## Abstract

Hypertension requires long-term therapy. However, antihypertensive drugs may cause adverse drug reactions (ADRs) affecting compliance and quality of life. This 12-month prospective observational study included 500 patients receiving at least one antihypertensive medication. A total of 95 ADRs (17.6%) were identified, most commonly due to calcium channel blockers and ACE inhibitors, with pedal edema, dry cough and dizziness being the frequent events. ADRs were significantly higher in patients on polypharmacy (p = 0.004) and most were classified as moderate and probable. Active pharmacovigilance is essential to improve safety and optimize hypertension management.

## Background:

Hypertension, also known as an elevated blood pressure, is the most significant risk factor of cardiovascular diseases, including stroke, heart attack and chronic kidney disease, on a global scale and this is a tremendous worldwide health issue [1]. The World Health Organization estimates that the 1.28 billion people with hypertension are concentrated in the low- and middle-income countries [2]. Long-term pharmaceutical intervention is also vital in hypertension treatment since it is necessary to maintain target blood pressure levels. The selection of drugs used to treat hypertension includes a wide range of drugs, among which are: diuretics, beta-blockers, calcium channel blockers, angiotensin-converting enzyme inhibitors, angiotensin II receptor blockers and many more [3]. Adverse drug reactions (ADRs) are typical of the use of these medicines, although it has been demonstrated to reduce the risk of cardiovascular disease. The World Health Organization (WHO) has defined adverse drug reactions (ADRs) as any harmful and unanticipated response to a drug that arises in situations when it is used in human medicine under normal doses to prevent, diagnose, or treat a disease [4]. These reactions can be as mild as such effects as nausea or vomiting and as deadly as angioedema or electrolyte imbalance. The effects of ADRs are complex and can cause severe impairments in the quality of life of a patient, poor adherence to medication, higher healthcare use due to the need to visit a physician more and even hospitalization and, finally, inadequate blood pressure control [5]. ADRs are seen to add millions of dollars to healthcare systems and cause a significant number of hospital admissions [6]. Hence, pharmacovigilance is the study and practice of identifying, measuring, perceiving and averting drug-related issues, hence constituting a base of reasonable interventions [7]. The recent research works on different geographical areas have reported the occurrence and trends of ADRs to antihypertensives, showing differences according to the prescribing habits, patient genetics and comorbidity [8, 9]. Indicatively, a high incidence of CCB-induced pedal edema has been documented in research in an Indian population and studies in Western populations have tended to examine the effects of ACEI-induced cough [10]. Despite this growing body of evidence, a significant research gap remains in the form of continuous, region-specific data collection within routine clinical practice. Many existing studies are retrospective or rely on spontaneous reporting systems, which are prone to under-reporting. Prospective observational studies provide a more accurate picture of ADR incidence and causality in a naturalistic setting. Therefore, it is of interest to prospectively detect and describe the prevalence, clinical pattern, causation, severity and preventability of adverse drug reactions (ADRs) linked to antihypertensive medications in patients undergoing treatment at a tertiary care hospital.

## Materials and Methods:

## Study design and setting:

Starting in January 2024 and ending in December 2024, this prospective, single-center, observational pharmacovigilance research tracked participants in the Department of Pharmacology, ESIC Medical College and Hospital, Bihta, Patna, Bihar, India, for a year.

## Study population and sample size:

The study population comprised adult patients diagnosed with essential hypertension. A sample size of 500 patients was enrolled using a convenience sampling method, based on the average patient load and feasibility within the study timeframe.

## Inclusion and exclusion criteria:

All participants had to meet certain criteria: they had to be at least 18 years old, diagnosed with hypertension and taking an antihypertensive medication for at least one month. Written informed permission was obtained from all individuals. Exclusion criteria for this research included the following: being pregnant or nursing, having a significant cognitive impairment, having trouble communicating, or being a part of another interventional medication trial.

## Data collection procedure:

Eligible patients were identified and recruited during their routine follow-up visits. After obtaining informed consent, a pre-designed data collection form was used to record patient information. This included demographic details (age, sex), anthropometric measurements (height, weight), social history (smoking, alcohol), clinical data (duration of hypertension, comorbidities) and a complete medication history (names of drugs, dose, frequency, duration). Direct interviews during follow-up visits and electronic medical record reviews were used to aggressively monitor patients for adverse drug reactions. In this study, adverse drug reactions (ADRs) were defined as any serious health problem a patient had while taking an antihypertensive medication, even if there was no direct link between the two. Extensive documentation was made of all suspected adverse drug reactions (ADRs), including the following: reaction description, date of start, treatment and outcome.

## ADR assessment tools:

We used the Naranjo causation Assessment Scale to determine the degree of causation for each possible ADR. This ten-item scale classifies ADRs as Definite (≥9), Probable (5-8), Possible (1-4), or Doubtful (0) based on the overall score. According to Hartwig and Siegel's Severity Assessment Scale, the reported ADRs were rated according to their severity. In this study, we used a scale that categorizes ADRs into three levels: mild (Level 1-2; no therapy change was necessary), moderate (Level 3-4; therapy change, specific treatment, or an increase of at least one day in the hospital were all necessary) and severe (Level 5-7; permanent harm, life-threatening, or death contributed).

## Statistical analysis:

We used SPSS for Windows, version 25.0, to analyze the data that was input into Microsoft Excel. To summarize the data, descriptive statistics were used. Categorical data were shown as frequencies and percentages (%), whilst continuous variables were shown as mean ± standard deviation (SD). The occurrence of adverse drug reactions in monotherapy compared to polypharmacy was one example of a categorical variable that was examined using the chi-square (χ^2^) test. For statistical purposes, a p-value below 0.05 was deemed significant.

## Results:

The research had 500 participants who were eligible to participate. Table 1 (see PDF) displays the demographic and clinical variables that were collected at baseline. People who took part in the study had an average age of 60.5 ± 11.2 years. There was a little male majority (54.0%) in the study population. The most common comorbidities were diabetes mellitus (35.2%) and dyslipidemia (28.8%). Out of 500 patients, 290 (58.0%) were on monotherapy, while 210 (42.0%) were on polypharmacy (receiving two or more antihypertensive drugs). Overall, 17.6% of patients had adverse drug reactions (ADRs) during the course of the research, with 88 individuals reporting a total of 95 ADRs. Table 2 (see PDF) displays the adverse drug reaction distribution by drug class that was involved. At 31.6%, calcium channel blockers were the most prevalent class of drugs linked with adverse drug reactions (ADRs), followed by angiotensin-converting enzyme (ACE) inhibitors at 25.3% (24 of 95 ADRs). The most frequently reported individual ADRs were pedal edema (23.2%), predominantly linked to amlodipine use, followed by dry cough (18.9%), which was almost exclusively associated with ACE inhibitors (ramipril and lisinopril). Dizziness (14.7%) was a common non-specific complaint associated with multiple drug classes, including diuretics and ARBs. "A statistically significant association was observed between the use of ACE inhibitors and the occurrence of dry cough (χ^2^ = 35.8, p < 0.001). As shown in Table 3 (see PDF), the Naranjo causality assessment revealed that the majority of ADRs were 'probable' (62 of 95; 65.3%), followed by 'possible' (29 of 95; 30.5%). Only four ADRs (4.2%) were classified as 'definite'. According to the Hartwig and Siegel scale, most ADRs were of moderate severity (54 of 95; 56.8%), requiring modification of therapy. Mild ADRs constituted 38.9% of the total, while 4.2% were severe. The most common management strategy was the withdrawal of the suspected drug (48.4%), followed by dose reduction or addition of a corrective drug (29.5%).

## Discussion:

In a tertiary care context, this prospective observational research sheds light on the prevalence and features of adverse drug reactions (ADRs) linked to antihypertensive drugs. The overall incidence of ADRs in our study was 17.6%, a finding that is consistent with the range reported in similar pharmacovigilance studies, which typically varies from 10% to 25% depending on the study design and population [11, 12]. This incidence rate underscores that ADRs are a common clinical problem in the long-term management of hypertension. The research we performed has revealed that CCBs and ACEIs are the most frequently associated classes of medications, which are associated with adverse drug reactions. This is agreeable to several other studies, as well as can be explained by their high rate of use, as well as their established side-effect profiles [10, 13]. Pedal edema and CCBs, especially amlodipine, were the most frequently encountered ADR. It is a documented side effect that is dose-dependent, resulting from arteriolar vasodilation and then fluid extravasation [14]. The second ADR was a persistent dry cough in relation to ACEIs. This has been confirmed by the strong statistical correlation in our study (p < 0.001), which demonstrates that there is a strong statistical association between substance P and bradykinin and drug-event associations. Clinicians need to be aware of these class-specific ADRs to provide adequate intervention, like switching an ACEI to an ARB, in a patient experiencing an intractable cough [15]. An important conclusion made during our study was that the rates of ADRs were found to be significantly higher in the group of patients who were on polypharmacy than in the group who were put on monotherapy (24.3% vs. 12.8%). This is a key point of concern because a significant number of hypertension patients, particularly those with comorbid conditions like diabetes, may need numerous agents to meet the target of blood pressure [3]. It is possible to explain the elevated risk associated with polypharmacy by a greater possibility of drug-drug interactions, additive drug effects, as well as a higher overall drug burden to the patient [16, 17]. To decrease the risks of adverse drug reactions (ADRs), it is essential to maintain rational prescription, regular medication review and deprescribe unnecessary drugs. The severity and causality assessments further placed the ADRs observed in the context of their therapeutic value. Likely and probably responses were the most frequent ones and usually they involved a need for change in therapy, either an alternative drug or a lower dose. This shows that the vast majority of ADRs that happened in our cohort were not insignificant complaints but actually had a physical effect on the patient's treatment. These ADRs must be managed as soon and as adequately as possible to promote adherence and continuance of effective antihypertensive treatment [5]. The strengths of this study are that it is designed prospectively; hence, it reduces bias related to recall and it can be actively monitored. The Naranjo algorithm and the Hartwig and Siegel scale are the standardized and validated scales of ADR assessment, which contribute to the strength of our results. Nevertheless, the research does not have only advantages. The results might not apply to other healthcare facilities, diverse populations of genetic origin, or prescription practices, as the study was carried out within a single facility. Furthermore, due to the limited number of the sample, our study might not have covered very rare ADRs.

## Conclusion:

The significance of pharmacovigilance to daily clinical practice is shown. Medical professionals ought to be highly suspicious of ADRs, counsel patients about their side effects and put timely measures in place to enhance the safety of patients, increase medication compliance and eventually record better long-term results in the treatment of hypertension.

## References

[1] Khurshid F (2012). Daru..

[2] https://www.who.int/.

[3] https://www.ncbi.nlm.nih.gov/books/NBK554579/.

[4] Geer MI (2016). J Pharmacol Toxicol Methods..

[5] Olowofela AO, Isah AO (2017). Ann Afr Med..

[6] Singh H (2010). J Young Pharm..

[7] Palaniappan M (2015). J Clin Diagn Res..

[8] Singh P (2020). J Evid Based Med Healthc..

[9] Hong D (2025). Drug Des Devel Ther..

[10] Kirilochev OO (2015). Int J Risk Saf Med..

[11] Aqil M (2012). J Pharm Bioallied Sci..

[12] Khan LM (2012). Saudi Med J..

[13] Chikkamath V (2025). Int J Pharma Clin Res..

[14] Richa (2015). Indian J Med Microbiol..

[15] Nasso C (2020). Front Pharmacol..

[16] Yan Z (2025). Front Pharmacol..

[17] Wolff J (2021). Pharmacoepidemiol Drug Saf..

